# Rapid on-site detection of harmful algal blooms: real-time cyanobacteria identification using Oxford Nanopore sequencing

**DOI:** 10.3389/fmicb.2023.1267652

**Published:** 2023-11-01

**Authors:** Marianne Potvin, Jeff Gauthier, Christophe Langevin, Vani Mohit, Naíla Barbosa da Costa, Thomas Deschênes, Maude Pomerleau, Irena Kukavica-Ibrulj, Daniel Verreault, Jérôme Comte, Roger C. Levesque

**Affiliations:** ^1^Institut de biologie intégrative et des systèmes (IBIS), Université Laval, Québec, QC, Canada; ^2^Centre Eau Terre Environnement, Institut national de la recherche scientifique (INRS), Centre Eau Terre Environnement, Québec, QC, Canada; ^3^Groupe de recherche interuniversitaire en limnologie (GRIL), Montréal, QC, Canada; ^4^Direction générale de la coordination scientifique et du Centre d’expertise en analyse environnementale du Québec (CEAEQ), Ministère de l’Environnement et de la Lutte contre les changements climatiques, de la Faune et des Parcs (MELCCFP), Québec, QC, Canada

**Keywords:** cyanobacteria, harmful algal blooms, on-site detection, Oxford Nanopore technologies, whole genome sequencing, cyanobacteria genomic database

## Abstract

With the increasing occurrence and severity of cyanobacterial harmful algal blooms (cHAB) at the global scale, there is an urgent need for rapid, accurate, accessible, and cost-effective detection tools. Here, we detail the RosHAB workflow, an innovative, in-the-field applicable genomics approach for real-time, early detection of cHAB outbreaks. We present how the proposed workflow offers consistent taxonomic identification of water samples in comparison to traditional microscopic analyses in a few hours and discuss how the generated data can be used to deepen our understanding on cyanobacteria ecology and forecast HABs events. In parallel, processed water samples will be used to iteratively build the International cyanobacterial toxin database (ICYATOX; http://icyatox.ibis.ulaval.ca) containing the analysis of novel cyanobacterial genomes, including phenomics and genomics metadata. Ultimately, RosHAB will (1) improve the accuracy of on-site rapid diagnostics, (2) standardize genomic procedures in the field, (3) facilitate these genomics procedures for non-scientific personnel, and (4) identify prognostic markers for evidence-based decisions in HABs surveillance.

## 1. A brief survey of cyanobacteria and algal bloom detection

Cyanobacteria are a diverse group of photosynthetic bacteria encompassing 1,600 species that can form dense and sometimes toxic blooms in freshwater and marine environments, and thereby represent a threat to natural ecosystems and water quality (Rastogi et al., 2015; Huisman et al., 2018). The toxic cyanobacterial blooms (cHABs) are of particular concern to human and animal health. In Canada, the increased occurrence of cHABs has been ascribed to climate change, habitat alterations from invasive species, overfishing and other human activities such as agricultural practices (Pick, 2016). A survey of cyanotoxin concentrations in 246 lakes revealed that all the Canadian provinces contained several lakes where toxin concentrations exceeded drinking water quality and sometimes recreational quality guidelines (Orihel et al., 2012). In Canada’s largest province, Quebec, 107 blooms per year on average have been reported between 2006 and 2016 (Ministère de l’Environnement et de la lutte contre les changements climatiques, 2018) and this number is certainly higher as not all blooms are reported to concerned authorities (Rashidi et al., 2021). Major issues linked to toxic cyanobacterial blooms have been identified in Quebec’s lakes within agricultural influenced watersheds (Missisquoi Bay: Fortin et al., 2010, 2015), in recreational lakes (Lajeunesse et al., 2012; Hudon et al., 2016; Lévesque et al., 2017) and in drinking water reservoirs (Lake Saint-Charles: Rolland et al., 2013).

With the increasing frequency of these cyanobacterial blooms, there is an urgent need to have early diagnostic tools for the detection and identification of cHABs in waterbodies. Several early warning tools have been developed to alert stakeholders by providing information on their origin, risk on drinking water contamination, toxicity levels and mitigation strategies. These tools include microscopic enumeration, pigment extraction and measurement, qPCR, toxin analysis and remote sensing as well as emerging techniques including next-generation sequencing, photonic systems, biosensors, drones, and applications of machine learning (Stauffer et al., 2019; MacKeigan et al., 2022).

Recently, a Canadian *Ecobiomics* (i.e., ecological microbiome metagenomics) strategy has been implemented to characterise aquatic biodiversity, to assess and monitor the health of aquatic ecosystems and to improve remediation strategies (Edge et al., 2020). This strategy provides useful tools for environmental monitoring, but it requires hiring highly qualified personnel for both laboratory and bioinformatics analyses as it relies on metabarcoding and shotgun metagenome sequencing, which makes it costly. It is not an easily transferable strategy that could be operated by non-scientific staff for on-site environmental diagnostics.

Nevertheless, the use of genomic tools is of particular interest in HABs monitoring since recent fast throughput genomics-based technology development indicates its application in the field at acceptable costs. Examples include metabarcoding time-series of cyanobacterial dynamics (Tromas et al., 2017), real-time DNA barcoding in rainforests (Pomerantz et al., 2018), workflows for tracking species diversity in the field (Maestri et al., 2019), metagenomics analysis of planktonic riverine microbial consortia (Reddington et al., 2020), metabarcoding analyses of algal and cyanobacteria assemblages to monitor biodiversity and ecosystems health (Ivanova et al., 2022; MacKeigan et al., 2022). Hence, there is a rational basis for monitoring cHABs and assess public health risks based upon the current fast-throughput genomics-based techniques (Te et al., 2015; Pérez-Carrascal et al., 2021; Urban et al., 2021; Yuan and Yoon, 2022).

## 2. A caveat for genomic-based identification: data scarcity

One of the main limitations in using genomic-based technologies for cyanobacteria identification is the lack of reference databases, which often cause incongruence of taxonomic assemblages obtained from microscopy and metabarcoding analyses (MacKeigan et al., 2022 and references therein). Current data resources for cyanobacterial research include a collection of at least 19 databases incorporating strain collections, genomics, proteomics, transcriptomics, regulatory information, descriptions of secondary metabolites, taxonomy and literature (Ramos et al., 2017; Kumar and Arya, 2020; Jones et al., 2021). However, these databases include only a few cyanobacterial species isolated Indeed, of all the 398,132 prokaryotic genomes currently on the NCBI genome database (as of July 26, 2023), only 3,552 (less than 1%) are from cyanobacteria.

Consequently, for a rapid analysis in real time and on-site, there is a critical need for a representative database of cyanobacteria, especially from freshwater ecosystems. However, one such database must be implemented quickly so that any novel rapid detection scheme can in turn use the added value of a specialized and high-quality annotated cyanobacterial database.

## 3. The rapid on-site detection of harmful algal blooms (RosHAB) strategy

We developed a cost effective on-site rapid detection of cyanobacterial strains that we refer to as RosHAB (Rapid on-site detection of Harmful Algal Blooms). The major value propositions of the RosHAB strategy are: (1) it is an on-site genomic technology in the form of a light mobile laboratory platform (see Supplementary Methods and Supplementary Figure S1) that will allow rapid identification of toxigenic cyanobacterial taxa, allowing environmental stakeholders to save analytic time and increase their analytic capacity for environmental monitoring of freshwater bodies. The duration between sample collection and delivery of results will be a few hours, compared to days when doing microscopic identification *ex-situ*; (2) It is an easy-to-use tool encompassing on-site collection, sample preparation, DNA extraction and sequencing of samples, together with a user-friendly bioinformatic platform. Short and accessible training will be needed before non-scientific staff can use the on-site tool as the DNA extraction method and DNA loading on the sequencing device will be straightforward and will not require complex laboratory skills.

**Figure 1:**
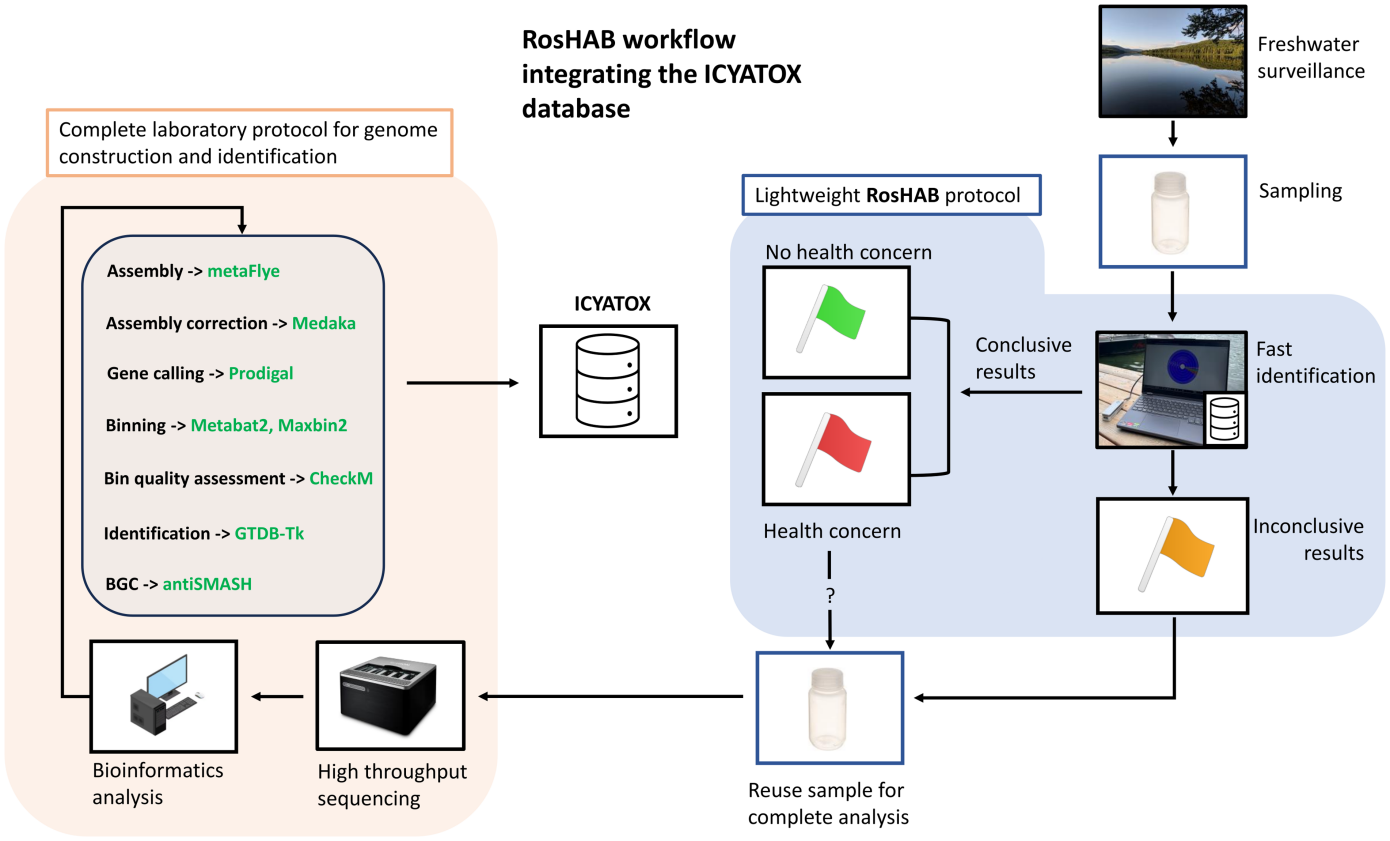
Overview of the RosHAB workflow and its integration with the ICYATOX database. Green flag: conclusive results without any public health concerns. Red flag: conclusive results with public health concerns. Some samples may require additional validation steps at the laboratory. Yellow flag: inconclusive results, further analysis needed. GridION photo: https://store.nanoporetech.com/gridion.html (last accessed Sept. 17th, 2023); computer photo by iStockPhoto user Makstorm (CC-BY 2.0). Nalgene(R) Plastic bottle photo: https://www.fishersci.ca. All other clip arts and photos were made by the authors.

The proposed genomic-derived solution provides the tools necessary to monitor in real time hundreds of waterbodies located in freshwater surveillance networks in the province of Quebec that eventually could be applied to other Canadian provinces. The flowchart in Figure 1 shows the RosHAB strategy for sampling and on-site samples processing, which includes preparation of genomic DNA, metagenome sequencing and analysis on a laptop using the Oxford Nanopore MinION portable sequencing system. When an environmental sample yields concerning results, the sample is reanalysed in a complete, high throughput metagenomics workflow to populate the International cyanobacterial toxin database (ICYATOX; www.icyatox.ca) with novel cyanobacterial genomes, including phenomics and genomics metadata (see section 4). This in turn reinforces the accuracy of RosHAB’s primary reference database (ICYATOX) as well as generating useful data for researchers and environmental stakeholders.

In an initial experiment, we compared the data depicted in Supplementary Table S1 from an unknown lake (hereby called Lake 1) as a blind assay. This lake was sampled once at the surface and results after 30 min versus 24 h sequencing runs were compared. Supplementary Table S2 shows results from a similar comparative study from a second unidentified lake (hereby called Lake 2), which was sampled in January (T1) and February 2022 (T2) at 3 different depths (1 sample each at 0.5 m and 12.5 m and duplicate samples at 7 m).

For each lake, we performed either a full-length sequencing run or a 30-min sequencing run. Furthermore, for Lake 2, we also compared two sequencing Oxford Nanopore systems: the conventional MinION (R9.4.1) flow cell, capable of generating an average of 20–25 Gigabases (Gb) of DNA sequencing data after 72 h, and a smaller less expensive version called the Flongle, which generates approximately 1 Gb of data after 24 h. Taxonomy was assigned to reads using the kmer-based classifier Kraken2 v2.1.2 (Wood et al., 2019) with minikraken_v2-2023-03^1^ as a reference database. To further demonstrate the portability of Nanopore sequencing and analysis, all analyses described above were run on an ASUS laptop computer with 16 GB RAM, 8-core Intel CPU and a NVIDIA GeForce GTX 980 M graphics card (the latter required to perform real-time base calling on the MinION). An in-house wrapper for Kraken2 coded in Bash was developed to produce taxonomic reports in HTML format at every 5 min interval, given that the minikraken reference database is fully loaded in RAM at runtime. Time estimates for this workflow have been included in Supplementary Table S3.

**Figure 2:**
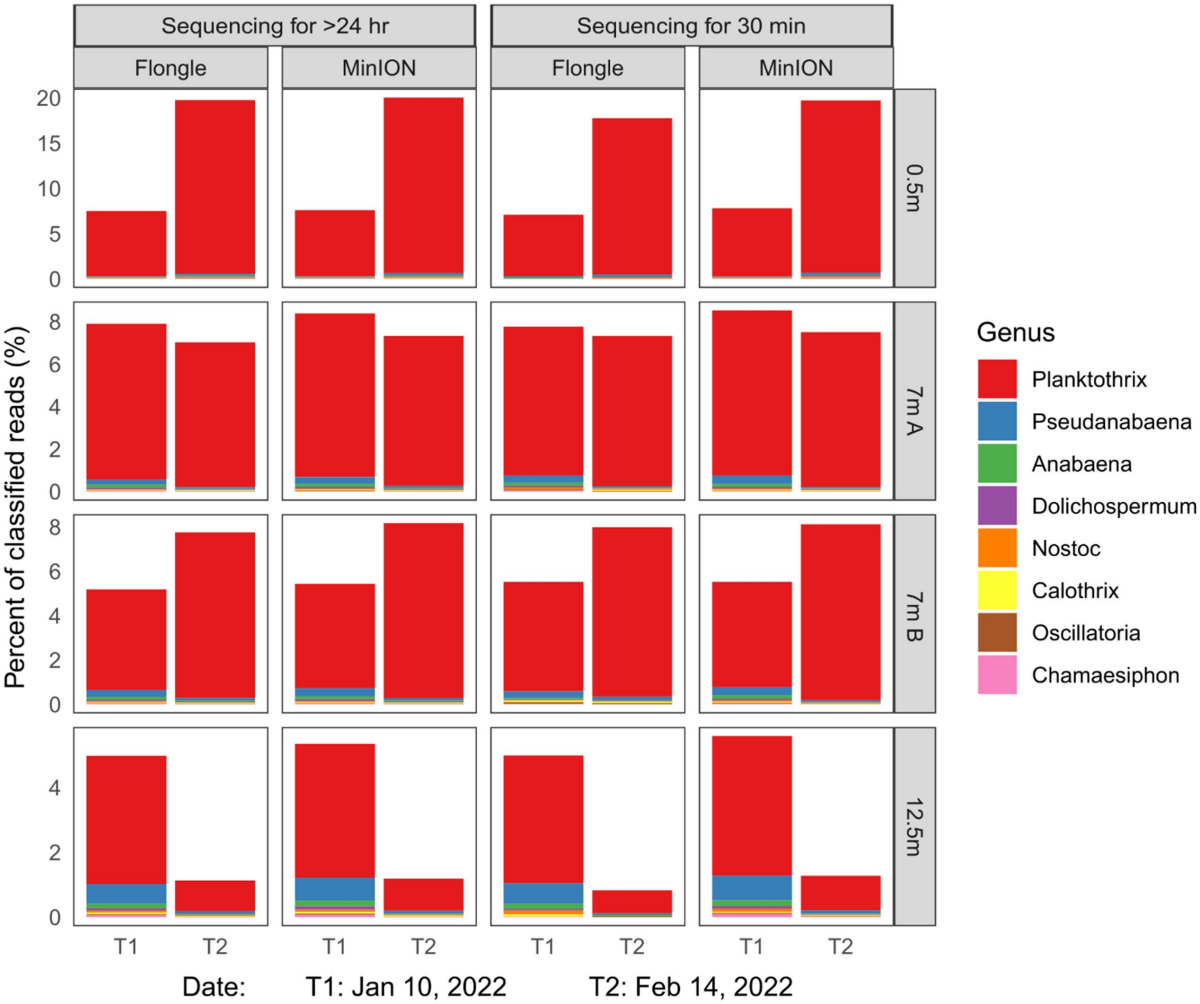
Relative abundance of the eight most abundant cyanobacterial genera in Lake 2, QC, Canada in different water depths, as measured with different sequencing throughputs (Flongle vs. MinION flow cell) and duty times (30 min vs. full run). * A complete sequencing run corresponded to 72 h for a flow cell and 24 h for a Flongle. Reads were base called and de-multiplexed with MinKNOW v21 (Oxford Nanopore). Reads less than 1,000 bp and average Phred score below 9 were discarded. For each sample, cyanobacterial relative abundance profiles were generated with Kraken2, a kmer-based taxonomic classifier (Wood et al., 2019). A lightweight version of the Kraken2 standard database (Minikraken, 8GB, version 20,200,312), was used to ensure low memory usage and runtime speed. Relative abundance charts and tables were built from Kraken2 output reports with R (R Core Team, 2021) and R package ggplot2 (Wickham, 2016).

Regardless of either run time (30 min vs. complete run) or sequencing depth (Flongle vs. MinION), similar abundance profiles were found (Supplementary Table S1 for Lake 1, Supplementary Table S2 for Lake 2). We identified cyanobacterial sequences up to the genus level using 17,200 reads from a Flongle versus 7 million from a flow cell. As shown in Supplementary Table S1, additional analyses in real-time on a laptop confirmed that the data obtained from a Flongle after 30 min of DNA sequencing and analysis was equivalent to a Flongle analysis done for 24 h. The Flongle yielded 91.8% identified reads after 30 min versus 92.9% after 24 h. Noteworthy, the suitability and consistency for clear species identification was maintained even when taxonomic resolution increased. In Lake 2, an algal bloom dominated by *Planktothrix* was confirmed after 30 min on a Flongle, consistent with a 24 h Flongle run and a 72 h MinION flow cell run (Figure 2 and Supplementary Table S2).

Overall, the dominant cyanobacterial genera in Lake 2 were, in order of relative abundances, *Planktothrix, Pseudanabaena, Anabaena, Dolichospermum, Nostoc, Calothrix, Oscillatoria,* and *Chamaesiphon* spp. Together, those eight taxa accounted for 2 to 20% of total reads, depending on sampling time and water depth (Figure 2). *Planktothrix* spp. dominated the surface and mid-depth and increased in relative abundance over the bloom period. Conversely, at 12.5 m *Planktothrix* relative abundance decreased during the same period (Figure 2). Regardless of the sequencing strategy and depth analyzed, standard deviation in the relative abundance profiles represents less than 1% (Supplementary Table S2). A chi-squared test of independence also corroborated the consistency of abundance measurements (0.8 < *p* < 0.93). Microscopic analyses of the samples further confirmed the taxonomic identification of the microbial DNA detection and assemblages and the dominance of *Planktothrix agardhii*.

In summary, a 30-min Flongle sequencing run, which represented 0.5–1% of a full-length MinION (R9.4.1) flow cell run was sufficient to obtain reliable cyanobacterial relative abundance profiles. Collectively these preliminary results showed that RosHAB can offer similar results using a cost-effective device, and that similar results to microscopy can be obtained in a time-effective manner to the species level.

## 4. The international cyanobacterial toxin database (ICYATOX)

The ICYATOX database^2^ is not only a genome data repository, but also archives samples and cyanobacterial cultures. Indeed, the primary source of material for ICYATOX are the DNA extracts and sample metadata produced through RosHAB, but will also consider (a) metagenomic resequencing of bloom samples using a higher-throughput platform (e.g., Oxford Nanopore GridION and Illumina short reads); (b) Sanger resequencing of a marker gene (e.g., 16S rRNA gene), (c) concentrated biomass from bloom samples cryopreserved at −80°C and (d) cyanobacterial cultures provided by researchers and collaborators. Indeed, when a water body tests positive for the presence of cyanobacteria, the DNA extract would be resequenced at higher throughput using the Oxford Nanopore GridION and Illumina short reads to undergo a rigorous metagenomic assembly, genome reconstruction (binning) and taxonomy pipeline. Cultures would also be treated as metagenomes, due to the difficulty to grow them axenically, and the importance of associated bacteria for their growth (Gao et al., 2020).

Briefly, raw Nanopore data from an environmental sample processed through RosHAB are quality filtered with NanoFilt (De Coster et al., 2018) using minimum Q scores and read lengths of 9 and 1,000 bp, respectively. Then, filtered reads are assembled with metaFlye v2.9 (Kolmogorov et al., 2019), after which metagenomic assemblies are corrected with Medaka v1.7.3.^3^ Metagenome-assembled genomes (MAGs) are reconstructed by blind binning with MetaBAT2 (Kang et al., 2019); genome completeness is verified with CheckM (Parks et al., 2015) and finally, taxonomic assignment is done with GTDB-Tk v2 (Chaumeil et al., 2022) using as the most recent GTDB reference database (Parks et al., 2022). Annotations are done with Prokka (Seemann, 2014), with an additional annotation scheme for antibiotic and secondary metabolite synthesis genes (e.g., genes responsible for toxin production) with AntiSMASH v6.0 in bacterial mode, with all extra options checked (Blin et al., 2021).

The ICYATOX database implementation uses MySQL5 and a Web-based ZenD Framework to describe cyanobacterial strains while providing genomic information linked to their phenotypic characterization and environmental data of the source lake. ICYATOX holds information such as the isolate ID, researcher responsible for the isolation, date, sample geographical origin and environmental variables describing it, phenotypic data, DNA extraction, sequencing information, and genome assembly. In the short term, the data processing mentioned above will be automated so that a RosHAB user without bioinformatics training may add samples themselves.

## 5. Significance and future directions

On the one hand, MAGs produced from ICYATOX isolates will allow identifying genes responsible for within-species variability (accessory genes) in addition to those underpinning conserved traits (core genes) within strains of the main bloom-forming species. To this regard, GTDB-Tk and AntiSMASH provide added value to the genomics workflow, as the first one is a highly elaborated taxonomic identification pipeline based on four criteria: average nucleotide identity, class-level phylogenetic placement, and core gene multiple sequence alignment with class-level neighbour genomes (Parks et al., 2022); the second one is a metabolite synthesis gene cluster (mBGC) annotation algorithm that integrates hidden Markov models (HMM), gene presence/absence and even enzyme numbers to predict the end product of a mBGC (Blin et al., 2021). The expansion of the ICYATOX database will provide valuable guiding for Canadian authorities, and potentially benefit other international authorities as well, in addition to provide reliable references of cyanobacterial genomes to future research.

On the other hand, the application of phenotype microarrays (PM) using small volumes of cyanobacterial culturesin small microtiter plates can provide the opportunity to perform many parallel assays in compact space, with a rapid turnover and at a low cost (Bochner, 2009; Borglin et al., 2012). PM can expand knowledge of the cyanobacterial metabolic potential, which could presumably be involved in cHABs initiation and expansion (Mobberley et al., 2013). Phenotype microarrays will be used to assess the growth of cyanobacteria from the ICYATOX strain collection in culture using multi-well plates, with a different test component in each well, enabling the screening of the phenotypic characteristics in a large throughput system.

There is an urgent need to refine our capacity to predict, prevent and mitigate cHABs given the economic and health challenges associated with them. As discussed above, there are now different tools available to inform environmental stakeholders and the public on aquatic ecosystem health including cHABs. Yet, to obtain an integrative understanding of cHABs, early warning systems available nowadays need to combine information from diverse analytical tools (e.g., Gaget et al., 2017). Recently, Almuhtaram et al. (2021) introduced a three-tiers framework to build a comprehensive early warning system that groups monitoring tools by their analytical targets: 1- biological activity or algal biomass (e.g., Chlorophyll-a concentration), 2- cyanobacteria or cyanobacteria-related genes (e.g., next generation sequencing), and 3- cyanobacterial metabolite (e.g., toxins). We plan to combine historical data with prediction models in order to forecast bloom events: through the identification of the main promoters/ factors that led to cHABs in the past and the construction of prediction models, we can improve the ability to forecast bloom outbreaks. Hence RosHAB will not only be a real-time, reliable, accessible, and cost-effective tool for early detection of cyanobacteria forming cHABs but will also represent a “self-iterative” approach to develop novel integrative biology approaches for bloom prediction.

## Data availability statement

The datasets presented in this study can be found in online repositories. The names of the repository/repositories and accession number(s) can be found at: http://www.ncbi.nlm.nih.gov/bioproject/998834, PRJNA998834.

## Author contributions

MarP, JG, CL, VM, and IK-I performed the experiments and data. DV, JC, and RL analyzed the data and drafted the manuscript. MarP, JG, CL, VM, NC, TD, MauP, IK-I, DV, JC, and RL handled cyanobacterial strains, collected metadata, and revised the manuscript. All authors contributed to the article and approved the submitted version.

## Footnotes

^1^ https://benlangmead.github.io/aws-indexes/k2

^2^ http://icyatox.ibis.ulaval.ca

^3^https://github.com/nanoporetech/medaka
