# Supplementary material for: Rapid on-site detection of harmful algal blooms: real-time cyanobacteria identification using Oxford Nanopore sequencing
Source: Front Microbiol. 2023 Nov 1;14:1267652. doi: 10.3389/fmicb.2023.1267652 (PMC10646836; doi:10.3389/fmicb.2023.1267652)
Supplement: Supplementary file 1 [file Data_Sheet_1.docx]

**Supplementary Methods**

1. **Pre-field assays**

Water samples were sent as unknown bloom content for blind assays and used to prepare nucleic acids with a rapid extraction system. DNA was extracted with the QIAGEN DNeasy PowerSoil Pro Kit. Between 655 and 1000 ng of DNA was used to prepare Oxford Nanopore Libraries using the Rapid Sequencing Kit (SQK-RAD004). 100 ng of final library was loaded on a Flongle flowcell (R9.4.1) for 24 hours and 493 ng was loaded on a MinION flow cell (R9.4.1) for 72 hours. Both sequence devices were placed on a GridION with mux scan set at 3 hours. Sequencing data were extracted after 30 min and at the end of each sequencing run for analysis. The percentage was calculated using classified reads only. Even after 30 minutes of DNA sequencing with a low-throughput Oxford Nanopore apparatus (Flongle), measurements are significantly similar regardless of sequencing throughput (Flongle vs MinION flow cell) and duty time (30 min vs full run). * A full run corresponded to 72 hr for MinION flow cells and 24 hr for Flongle.

1. **Material for on-site detection using RosHAB (see Figure S1 below)**

Sampling kit:

50 mL syringe, Sterivex filter unit, Plastic tubing cutter, Filter forceps, Sterile Petri dish, Ethanol 95%, Nitrile gloves.

Extraction and library prep:

Minicentrifuge, DNeasy PowerSoil Pro Kit, Pipettes and Tips (2, 20, 200 and 1000 µL), Trash container (old pipette tips box), Microtube racks, Timer, Vortex mixer, Reagents from ONT Rapid Sequencing Kit (SQK-RAD004), QuantaBio SPARQ PureMag magnetic bead solution), Magnetic rack, Qubit dsDNA BR kit and fluorometer, Mini thermocycler (Eppendorf MiniPCR-8).

Sequencing:

- Laptop computer (64-bit AMD Ryzen CPU, 16 GB RAM, USB 3.0 connectivity, and NVIDIA graphics card (Geforce RTX series), MinION MkIB portable sequencer, Flongle adapter, Flongle flow cell.

**Suppl. Fig. 1.** Illustration of the RosHAB detection workflow in action, at an undisclosed location.


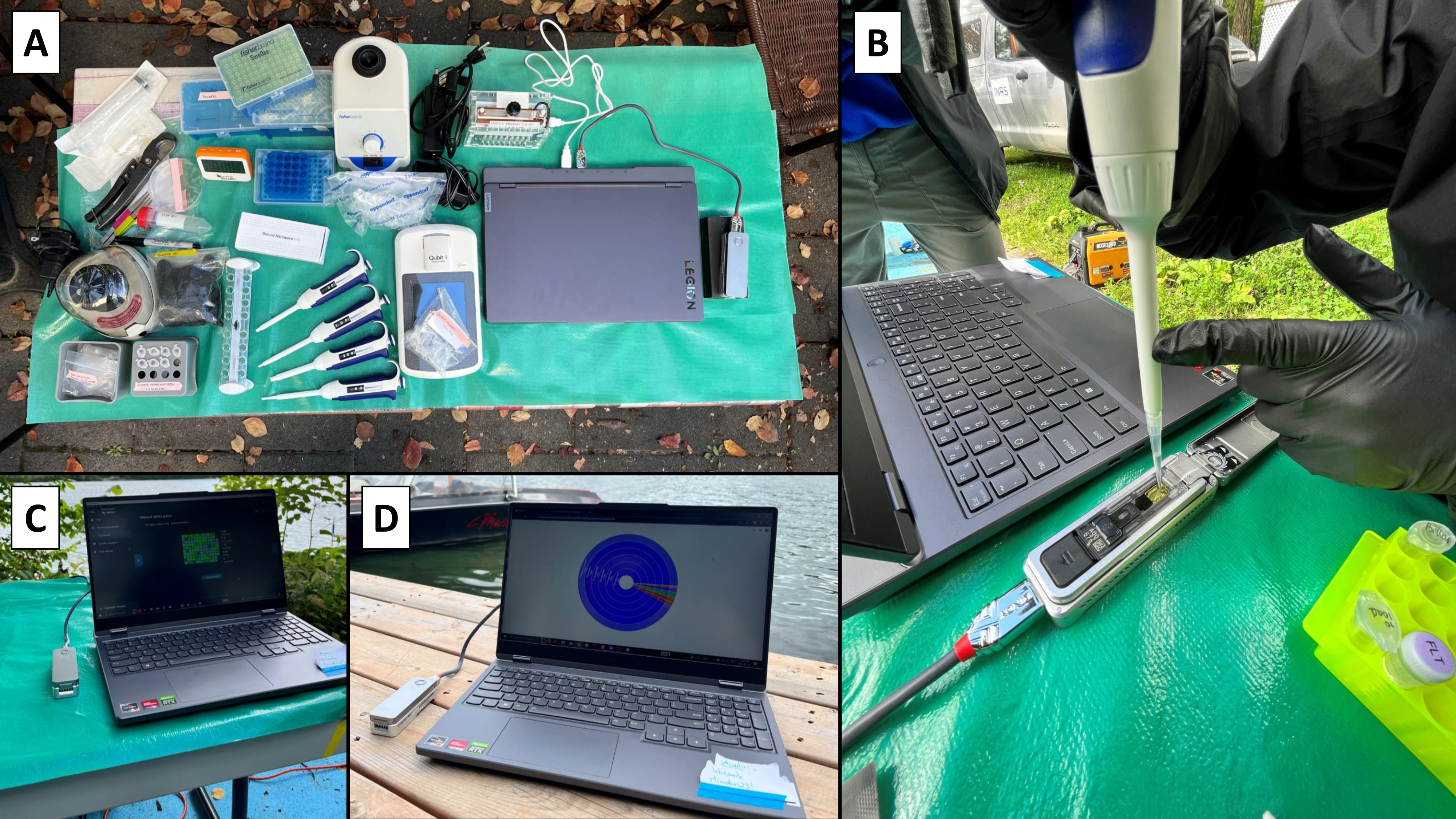


A: Mobile laboratory setup for sample collection and DNA extraction, which includes the QIAGEN PowerSoil Pro DNA Kit, Oxford Nanopore Rapid Sequencing Kit (SQK-RAD004) reagents and laboratory equipment as well. Time from sample collection to obtaining a sequencing library was 2 h 30. B: Loading a Flongle with a rapid sequencing library. C: Sequencing in progress. D: Obtaining a first taxonomic report 30 min after starting the sequencing run.

**Suppl. Table 1.** Analysis of a water sample from an unknown lake (Lake 1) and tested in a blind assay for bloom content.

|  | Flongle analyses for 30 min | | Flongle analyses for 24 h | |
| --- | --- | --- | --- | --- |
|  | Number of Reads | % | Number of Reads | % |
| DNA reads analyzed | 12 000 |  | 249 333 |  |
| Classified reads | 11 051 | 92.1 | 233 096 | 93.5 |
| Unclassified reads | 949 | 7.9 | 16 237 | 6.5 |
| Taxonomy | Number Reads | % Classified | Number reads | % Classified |
| Bacteria | 10 140 | 91.8 | 216 636 | 92.9 |
| Cyanobacteria | 9353 | 84.6 | 201 621 | 86.5 |
| Oscillatoriales | 9229 | 83.1 | 199 500 | 85.6 |
| *Planktothrix agardhii* | 9027 | 81.7 | 195 668 | 83.9 |

**Suppl. Table 2.** Relative abundance of *Planktothrix* spp. in Lake 2, QC, Canada at different water depths at 1 month interval.

| **Planktothrix (genus)** | | **% Abundance (30 min sequencing)** | | **% Abundance (>24 hr sequencing*)** | | **Standard deviation** | **CI lower (95%)** | **CI upper (95%)** | **X^2^ test P-value (30 min vs full run)** |
| --- | --- | --- | --- | --- | --- | --- | --- | --- | --- |
| **Depth** | **Date** | **Flow Cell** | **Flongle** | **Flow Cell** | **Flongle** |  |  |  |  |
| 0.5m | 2022-01-10 | 7.54 | 6.78 | 7.31 | 7.21 | 0.32 | 7.03 | 8.05 | 0.86 |
|  | 2022-02-14 | 19.04 | 17.27 | 19.41 | 19.18 | 0.98 | 17.48 | 20.60 | 0.66 |
| 7m (A) | 2022-01-10 | 7.77 | 7.01 | 7.7 | 7.33 | 0.35 | 7.21 | 8.33 | 0.90 |
|  | 2022-02-14 | 7.28 | 7.07 | 7.06 | 6.81 | 0.19 | 6.97 | 7.59 | 0.90 |
| 7m (B) | 2022-01-10 | 4.76 | 4.94 | 4.71 | 4.53 | 0.17 | 4.49 | 5.03 | 0.85 |
|  | 2022-02-14 | 7.93 | 7.64 | 7.88 | 7.45 | 0.22 | 7.58 | 8.28 | 0.94 |
| 12.5m | 2022-01-10 | 4.31 | 3.94 | 4.13 | 3.96 | 0.17 | 4.04 | 4.58 | 0.93 |
|  | 2022-02-14 | 1.06 | 0.71 | 0.99 | 0.94 | 0.15 | 0.82 | 1.30 | 0.80 |

**Suppl. Table 3.** Time estimates for all steps of the RosHAB detection workflows, based on the preliminary assays mentioned in Suppl. Table 1 and Suppl. Table 2.

| Step | Method | Estimated time |
| --- | --- | --- |
| Concentration | Filtration onto 0.22 um membrane using a syringe | 10-20 min  Volume-dependent |
| DNA extraction | Qiagen PowerSoil Pro DNA Kit | 1h |
| Library preparation | Rapid kit (single sample) | 30 min |
|  | Rapid kit (with barcoded samples) | 1h |
| Sequencing | Flongle on MinION Mk1B | 30 min (minimum) |
| Taxonomic assignment | Kraken2 (on laptop) | 3 min 2 sec |
